# Resveratrol-loaded PLGA nanoparticles: enhanced stability, solubility and bioactivity of resveratrol for non-alcoholic fatty liver disease therapy

**DOI:** 10.1098/rsos.181457

**Published:** 2018-11-14

**Authors:** Shuqian Wan, Long Zhang, Yunyun Quan, Kun Wei

**Affiliations:** 1 School of Biological Science and Engineering, South China University of Technology, Guangzhou 510640, People's Republic of China; 2 Wenzhou Institute of Biomaterials and Engineering, CAS, Wenzhou, Zhejiang 325011, People's Republic of China

**Keywords:** resveratrol, PLGA nanoparticles, stability, solubility, bioactivity, non-alcoholic fatty liver disease

## Abstract

Resveratrol (3, 4′, 5-trihydroxy-*trans*-stilbene, RSV), a nutraceutical, has recently attracted lots of attention because of its outstanding pharmacological potential. The effects of RSV on non-alcoholic fatty liver disease (NAFLD) remain inconclusive, although a wealth of research has been done. The major obstacle presented was RSV's poor bioavailability due to its poor aqueous solubility, chemical instability and intestinal metabolism. In this study, nanotechnology was used to encapsulate RSV to enhance its stability, water solubility and bioactivity, which can be used to treat NAFLD by HepG2 hepatocytes-induced *in vitro*. RSV-loaded poly (d, l-lactide-co-glycolide acid) (PLGA) nanoparticles (RSV-PLGA-NPs) were prepared according to an oil/water (O/W) emulsion technique. The RSV-PLGA-NPs were of spherical morphology with an average size of 176.1 nm and a negative charge of −22.6 mV. These nanoparticles exhibited remarkable encapsulation efficiency (EE%) (97.25%) and drug loading (14.9%) for RSV. A sustained RSV release from RSV-PLGA-NPs could be achieved especially in acidic conditions when simulating transporting through the gastrointestinal tract. In addition, these nanoparticles were stable enough to store at 4°C for a least six months with unchanged EE%. Moreover, RSV-PLGA-NPs were more efficient in alleviating lipogenesis, promoting lipolysis and reducing hepatocellular proliferation than free RSV due to its improved stability, water solubility and bioactivity. These findings indicated that the RSV-PLGA-NPs provided superb and stable drug delivery with small particle size, high capsulation efficiency, well-controlled drug release, which greatly enhanced the stability, water solubility and bioactivity. Besides, the discovery that the inhibitory effect of RSV-PLGA-NPs on hepatocellular proliferation and lipid accumulation in steatotic HepG2 cells may provide a new way to study the mechanism of NAFLD. Therefore, RSV-PLGA-NPs have a promising potential for NAFLD therapy.

## 1. Introduction

Non-alcoholic fatty liver disease (NAFLD) is the most prevalent chronic liver disease worldwide [1,2]. One sign of pathological findings is the excess triglyceride accumulations within hepatocytes (steatosis) [3]. It is closely associated with obesity, type 2 diabetes and metabolic syndrome [4]. Furthermore, NAFLD is rapidly growing to be the primary cause of liver failure and liver transplants [3,5]. However, there are few accepted pharmacologic therapies for NAFLD currently [6], and the mainstay of treatment for NAFLD presently is the adoption of lifestyle changes aimed at increased physical activity and moderate, sustained weight loss [7]. Studies have assessed: insulin-sensitizing agents (thiazolidinediones); anti-oxidants (vitamin E, hepatic iron reduction, betaine, S-adenosyl-methionine, N-acetyl cysteine and probucol); lipid-lowering agents (3-hydroxy-3-methylglutaryl-coenzyme A reductase inhibitors and fibrates) and cytoprotective agents (ursodeoxycholic acid) [6,7]. But these pharmacological therapies were lack of specificity and effectiveness [6–8]. So it remains an urgent need for improved pharmacological therapies.

Bioactive food constituents propose new treatment approaches in the modulation of some disease [9]. Resveratrol (3,4′,5-trihydroxy-*trans*-stilbene, RSV) has recently attracted lots of attention because of its outstanding pharmacological potential, including anti-oxidation, anti-inflammation, anti-cancer, treatment of diabetes, anti-obesity and so on [10–12]. It is a natural non-flavonoid polyphenol [13] widely found in plants such as grapes, knotweed and peanuts [1]. Biochemically, NAFLD is characterized by inactivation of AMPK, hepatic lipid accumulation, decreased insulin sensitivity and inflammation [14,15]. As RSV has been shown to decrease inflammation, activate SIRT1 and mimic the effects of caloric restriction, many researchers have predicted that resveratrol could be a potential treatment option for NAFLD. Though a wealth of research has been done, the effects of RSV on NAFLD remain inconclusive. Some preclinical studies have demonstrated a preventive role of resveratrol in NAFLD [16,17]. However, some other evidence does not support the opinion [2,18]. Given the contradictory results and limited experimental data available, many studies have to be done to prove its effectiveness, and the application of RSV as a treatment for NAFLD still has a long way to go.

The major obstacle presented was RSV's poor bioavailability [1], due to poor aqueous solubility, chemical instability and intestinal metabolism [19,20]. Existing researches about NAFLD therapy are almost based on bare RSV. This may be one of the reasons that the bioavailability of RSV is poor *in vivo*. So many concerns regarding its effectiveness arise from its poor stability *in vivo* and low bioavailability following oral ingestion [19]. Therefore, the stability of RSV *in vitro* should be paid more attention because the instability can decrease the bioavailability and bioactivity *in vivo*. Moreover, the stability of the RSV or its delivery system has not been studied in detail in other studies. Nanocarrier has rapidly received recognition for enhancing the bioavailability and solubility, improving the stability of drugs [21], especially for some highly hydrophobic drugs [22,23]. Wang *et al*. showed how attaching flexible lipid chains to cabazitaxel (a hydrophobic and highly toxic anti-cancer drug) can convert it to a systemic self-deliverable nanotherapy, preserving its pharmacologic efficacy while improving its safety profile [22]. Lin *et al*. used RSV-loaded nanoparticles conjugated with KIM-1 antibody as a novel method for potential use in chronic kidney disease [24]. Vijayakumar *et al*. applied poly PLGA–D-a-tocopheryl polyethylene glycol 1000 succinate blend nanoparticles (RSV-PLGA-BNPs) for brain cancer therapy [25]. Although there are some studies that have reported using nano-drug delivery technology to encapsulate RSV, few papers reported just using poly (d, l-lactic-co-glycolic acid) (PLGA) as a carrier to delivery RSV to treat NAFLD. PLGA has been approved by the US Food and Drug Administration (FDA) to be clinically applied for some anti-cancer drugs [25,26]. Because of the excellent biocompatibility and degradability in a physiological environment, controlled release [27,28] and its biodegradation products (lactic acid and glycolic acid) which are metabolites presenting in the human body, PLGA has been used as an effective carrier for drug delivery [21].

In this study, an optimized method for preparing PLGA nanocarriers (RSV-PLGA-NPs) was used to deliver RSV, aiming to enhance the stability, solubility and bioactivity *in vitro* to treat NAFLD. RSV-PLGA-NPs were characterized by particle size, zeta potential, poly-dispersity index (PDI), Fourier-transform infrared (FTIR) spectroscopy, morphology, encapsulation efficiency (EE%) and drug loading (DL%), release of RSV and degradation of RSV-PLGA-NPs *in vitro*. The formulation performances of RSV-PLGA-NPs were evaluated with cellular uptake, *in vitro* cytotoxicity assay, *in vitro* anti-fat accumulation, lipolysis and cell proliferation in oleic acid (OA)-induced HepG2 cells. The RSV-PLGA-NPs, with high EE% and DL% of RSV, had a strong stability to prevent isomerization of RSV and enhance its bioactivity. The inhibitory effect of RSV-PLGA-NPs on hepatocellular proliferation and lipid accumulation meant RSV had the promising pharmacological potential to treat NAFLD.

## 2. Material and methods

### 2.1. Materials

Poly (d, l-lactic-co-glycolic acid) (PLGA, molar ratio of d, l-lactic to glycolic acid, 50 : 50, MW = 31 kDa) was purchased from Jinan Daigang Biomaterial Co., Ltd (Shandong, China); bovine serum albumin (BSA, PH0501), phosphate buffer saline powder (PBS, PH1403) were acquired from Phygene; resveratrol (RSV, R8350-5 G) was purchased from Solarbio; Oil Red O (ORO, O1391-250 ML), Fluorescein isothiocyanate (FITC, F7250-100MG), 0.25% trypsin-EDTA were purchased from Sigma; OA (O1008-1 G) was purchased from Sigma-Aldrich. Dulbecco's Modified Eagle's Medium (DMEM) was purchased from Gibco; dimethyl sulfoxide (DMSO, D119415-1 L) was purchased from Aladdin; sodium dodecyl sulfate (SDS, S817788-500 g), paraformaldehyde (P804537-500G), isopropyl alcohol (I811925-500ML) were purchased from Macklin; dichloromethane, acetone were purchased from Guangzhou Jinhauda Chemical Co., Ltd. Foetal bovine serum (FBS) and penicillin–streptomycin solution were purchased from Gibco. MTT cell proliferation and cytotoxicity assay kit (PH0533) was obtained from Phygene; triglyceride assay kit (A110-1) was obtained from Nanjing Jiancheng Bioengineering Institute; glycerine enzyme assay kit (E1002) was obtained from Applygen Technologies Inc. BCA protein assay kit (C503021) was obtained from Sangon Biotech (Shanghai) Co., Ltd.

### 2.2. Cell culture

Human liver cancer cell line HepG2 was purchased from Procell Life Science & Technology Co., Ltd (Wuhan, China). HepG2 cells were cultured in high glucose DMEM supplemented with 10% FBS and 1% of penicillin/streptomycin at 37°C in 5% CO_2_. After reaching 80% confluence, the HepG2 cells should be subcultured. The subcultivation ration is 1 : 3. The cells used for the experiment were within 15 generations.

### 2.3. Preparation of RSV-PLGA-NPs

Fifty milligrams of PLGA was dissolved in 5 ml of dichloromethane and acetone (dichloromethane/acetone = 3/2) to form well-proportioned PLGA solution (10 mg ml^−1^) in which PLGA was dissolved completely. A certain amount of RSV (RSV was dissolved in ethanol) was added to the PLGA solution and sonicated at 200 W for 2 min to produce a primary emulsion (organic phase). Then the primary emulsion was slowly injected into the BSA solution (1% w/v) (aqueous phase) and sonicated at 200 W for 4 min again. The final oil/water (O/W) emulsion was made. To disperse the final O/W emulsion, 15 ml of deionized water was added and stirred magnetically for some hours to remove the residual organic solvent. After being centrifuged at 14 000 r.p.m. for 30 min, nanoparticles were obtained and the supernatant was removed. Then the collected NPs were washed with deionized water three times by centrifuging at 10 000 r.p.m. for 20 min. Finally, the NPs were resuspended with deionized water and then lyophilized by vacuum freeze dryer under −50°C. The freeze-dryed nanoparticles were stored at 4°C. We optimized the preparation method from the volume ratio of organic and water phase, the amount of RSV and the stirring time to obtain the maximum EE% and the optimum particle size.

### 2.4. Size and zeta potential

Particle size and zeta potential of PLGA-NPS and RSV-PLGA-NPs were determined by dynamic light scattering (DLS) using a Zetasizer Nano-ZS90 (Malvern Instruments, Worcestershire, UK). The nanoparticles were dissolved in deionized water and the concentration was 5 mg ml^−1^.

### 2.5. Scanning electron microscopy and atomic force microscope

The morphology and size of dry PLGA-NPS and RSV-PLGA-NPS were measured by field emission scanning electron microscopy (SEM) (HITACHI, SU8010, Japan) and atomic force microscopy (AFM) (Bruker, Dimension Icon, USA). 0.2 mg of nanoparticles was dissolved in 1 ml of deionized water. Then a drop of suspension of PLGA-NPS or RSV-PLGA-NPs was placed on an alcohol-treated clean wafer and dried at room temperature.

### 2.6. Encapsulation efficiency and drug loading

To measure the optimal dosing ratio, we evaluated the EE% and the DL% of RSV-PLGA-NPs with different amounts of RSV. One milligram of lyophilized RSV-PLGA-NPs was dissolved in 1 ml DMSO. In order to completely destroy the NPs structure, it was sonicated under 100% ultrasound for 10 min to ensure complete release of the encapsulated RSV. Then, the sample was centrifuged at 10 000 r.p.m. for 10 min. The supernatant was taken and diluted 50-fold with DMSO for measurement.

The modular multi-technology microplate reader (ThermoFisher, Varioskan LUX, USA) was used to measure the fluorescence intensity of the sample at an excitation wavelength (Ex) of 356 nm and an emission wavelength (Em) of 383 nm. The calibration curve of the DMSO solution of RSV was used to calculate the content of RSV in the corresponding sample.

$$\begin{array}{rl} \mathrm{EE}\text{\%}= & \frac{W_{1}}{W_{2}}\times 100\text{\%},\\ \mathrm{DL}\text{\%}= & \frac{W_{1}}{W_{1}+W_{3}}\times 100\text{\%}, \end{array}$$

where *W*_1_ is the amount of RSV encapsulated in RSV-PLGA-NPs, *W*_2_ is the total amount of RSV used for the preparation of nanoparticles and *W*_3_ is the total amount of PLGA used for the preparation of nanoparticles.

### 2.7. Stability study

The RSV-PLGA-NPs were kept in deionized water at 4°C and protected from light. And then NPs were assayed periodically, at the time points of zero, one, three and six months, for particle size, zeta potential, PDI and EE%. To detect the function of RSV-PLGA-NPs to protect the biological activity of RSV, RSV-PLGA-NPs were exposed to ultraviolet light for an hour before use.

### 2.8. Fourier-transform infrared spectroscopy

The structure and composition-related functional groups of the compounds were identified by infrared absorption spectroscopy (Bruker, Tensor II, Germany). The infrared spectra of naked RSV, PLGA-NPs and RSV-PLGA-NPs were measured with an attenuated total reflectance (ATR) accessory at a wavelength of 4000–400 cm^−1^. The RSV powder and lyophilized RSV-PLGA-NPs and lyophilized PLGA-NPs were used to be measured directly without special preparation.

### 2.9. Release kinetics *in vitro*

Eight milligrams of RSV-PLGA-NPs was aspirated in a dialysis bag (this operation was repeated three times). After that, the three dialysis bags were put into 150 ml of PBS solution at pH 1.2, 6.8 and 7.4, respectively, which are simulated gastric, intestinal and intravenous fluid, respectively. The different pHs of PBS solutions all contain 1.5% SDS (an anionic surfactant) to increase the solubility of RSV in water. The *in vitro* releasing was performed by continuous shaking at 37°C in a shaker at 100 r.p.m. One millilitre solution was sampled each time at predetermined time intervals (2, 8, 24, 48, 72, 72, 96, 120, 168, 192 and 216 h). And then 1 ml solution of the corresponding pH in PBS solution was supplemented. The modular multi-technology microplate reader (ThermoFisher, Varioskan LUX, USA) was used to measure the fluorescence intensity of the sample at an excitation wavelength (Ex) of 356 nm and an emission wavelength (Em) of 383 nm. Finally, the RSV content in the corresponding release solution was calculated using the calibration curve of RSV in PBS (containing 1.5% SDS).

$$\mathrm{cumulative}\;\mathrm{release}\;\text{\%}=\frac{W_{R}}{W_{T}}\times 100\text{\%},$$

Where, *W*_R_ is the total amount of RSV that had been released in the medium including being sampled every time, and *W*_T_ is the total amount of RSV encapsulated in RSV-PLGA-NPs.

### 2.10. Degradation kinetics *in vitro*

Six milligrams of RSV-PLGA-NPs was dispersed in 6 ml PBS with pH of 7.4, 6.8 and 1.2, respectively. Degradation kinetics *in vitro* was performed by continuous shaking at 37°C in a 100 r.p.m. shaker. The samples were measured at predetermined time intervals (1, 2, 4 h, and 1, 2, 3, 5, 7, 9, 13, 18, 22, 25, 27, 30 and 32 days). The pH of samples was measured by pH meter.

### 2.11. Cellular uptake

The pictures of cellular uptake were imaged with live cell workstation. The method of preparing FITC-labelled RSV-PLGA-NPs is the same as that of RSV-PLGA-NPs, just adding 200 µl of 1 mg ml^−1^ FITC (green fluorescence) together with RSV. HepG2 cells (5 × 10^4^ cells well^−1^) were seeded in 24-well plates. After attaching, cells were incubated with FITC-PLGA-NPs for 2 and 8 h. Then cells were incubated with DAPI (blue fluorescence for nuclear labelling) for 15 min. After staining with DAPI, the cells needed to be washed three times in PBS to remove excess dye.

### 2.12. *In vitro* cytotoxicity assay

The cytotoxicity of naked RSV, PLGA-NPs, RSV-PLGA-NPs and OA to HepG2 was measured by MTT assay. Briefly, HepG2 was seeded into 96-well plates at 1 × 10^4^ cells well^−1^. After adherence of cells, RSV, PLGA-NPs and RSV-PLGA-NPs were added to each well, respectively. They were incubated together with cells for 24 h. DMEM medium without FBS was used throughout. 10 µl of reagent A (MTT solution) was added to each well (100 µl medium). The cells were incubated for 4 h in a cell incubator at 37°C with 5% CO_2_. The supernatant was discarded, and reagent B (Formazan Solvent) was added to incubate with cells for 10 min. The absorbance was recorded at 570 nm by the modular multitechnology microplate reader (ThermoFisher, Varioskan LUX, USA). The specific operation steps were refered to MTT cell proliferation and cytotoxicity assay kit (PH0533).The control group of RSV, PLGA-NPs and OA was treated with DMSO, and the control group of RSV-PLGA-NPs was treated with PLGA-NPs.

### 2.13. Oleic acid-induced hepatic steatosis

Hepatic steatosis induced by OA was used as a non-alcoholic fatty liver model *in vitro*. HepG2 cells were inoculated into 12-well plates at 1 × 10^5^ cells well^−1^. To determine the appropriate concentration of OA, adherent cells were incubated with different concentrations of OA for 24 h to induce cell steatosis. The specific evaluation criteria were determined by Oil Red O staining. The control group of OA was treated with DMSO.

### 2.14. Oil Red O staining

In order to visually show the content of triglyceride (TG) in the cells, the method of Oil Red O staining was used. The cells were gently washed three times with PBS and fixed with 4% paraformaldehyde solution at room temperature for 15 min. Subsequently, the cells were washed three times with PBS to remove the remaining paraformaldehyde solution, and stained with freshly prepared Oil Red O dye (the volume ratio of Oil Red O to water was 3 : 2) working solution at room temperature for 30 min. Next, the cells were washed twice with 60% isopropanol to remove floating colour and then washed three times with PBS. Finally, the cells were infiltrated with PBS and photos taken under a microscope.

### 2.15. Quantitative assessment of triglycerides

To quantitatively detect the effect of RSV and RSV-PLGA-NPs on fat accumulation after OA induction, the triglyceride kit was used for the assay. After induction of OA, cells were incubated with RSV and RSV-PLGA-NPs for 24 h, respectively. The supernatant was discarded and the cells were digested. The accumulated triglyceride levels in the cells were measured according to the instructions of the triglyceride assay kit (A110-1) to determine the degree of fat accumulation. The control group of RSV was treated with DMSO, and the control group of RSV-PLGA-NPs was treated with PLGA-NPs.

### 2.16. Quantitative assessment of glycerol

In order to quantitatively detect the effect of RSV and RSV-PLGA-NPs on lipolysis after OA induction, a glycerol kit was used for the assay. After induction of OA, the cells were incubated with RSV and RSV-PLGA-NPs for 24 h, respectively. The medium used DMEM without serum and without phenol red. The supernatant was collected and was centrifuged at 12 000 *g* for 5 min. The glycerol content in the supernatant was measured according to the instructions of glycerine enzyme assay kit (E1002) to determine the degree of lipolysis. The control group of RSV was treated with DMSO, and the control group of RSV-PLGA-NPs was treated with PLGA-NPs.

### 2.17. Cell proliferation

The effect of RSV and RSV-PLGA-NPs on the proliferation of HepG2 cells before induction was measured by MTT cell proliferation and cytotoxicity assay kit (PH0533). Untreated cells were selected as controls. Briefly, HepG2 was seeded into 96-well plates at 1 × 10^4^ cells well^−1^. RSV and RSV-PLGA-NPs were added to each well, respectively. They were incubated together with cells for 24 h. DMEM medium containing 10% FBS was used throughout. The absorbance was recorded at 570 nm by the modular multi-technology microplate reader (ThermoFisher, Varioskan LUX, USA). The effect of RSV and RSV-PLGA-NPs on cell proliferation of HepG2 cells after induction was determined by BCA protein assay kit (C503021). After the induction of OA, cells were incubated with RSV and RSV-PLGA-NPs for 24 h. The supernatant was discarded and the cells were digested. The protein content determined according to the instructions of BCA protein assay kit (C503021) could represent the cell number. The control group of RSV was treated with DMSO, and the control group of RSV-PLGA-NPs was treated with PLGA-NPs.

### 2.18. Statistical analysis

The data were expressed as the mean ± standard deviation (s.d.). The significance of differences was assessed by one-way analysis of variance (ANOVA) followed by Tukey's multiple comparison tests (*t*-test). The data were considered significant when *p* < 0.05. Data analysis was carried out by GraphPad Prism v. 6.01 (GraphPad Software) and OriginLab OriginalPro v. 8.5.1.

## 3. Results and discussion

### 3.1. Preparation and characterization of NPs

The emulsified solvent evaporation method is a straightforward and facile preparative process of polymeric NPs [21,29]. PLGA has been widely used as a carrier of the delivery drug, due to its minimal systemic toxicity, biocompatible and biodegradable [26]. In this study, RSV was wrapped by PLGA as a carrier to enhance its stability, solubility and pharmacological potential. The formulation constitutions influence the formulation performance, such as particle size, EE% and stability of NPs.

**Figure 1.**
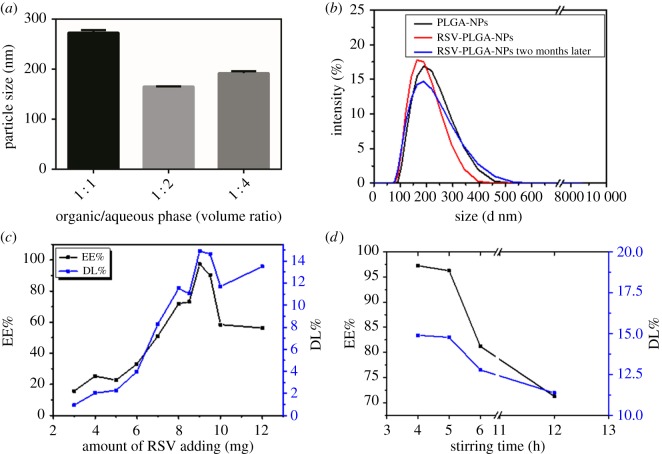
Factors influencing the formulation properties of RSV-PLGA-NPs. (*a*) 50 mg PLGA versus 8 mg RSV, 4 h of stirring time; (*b*) 50 mg PLGA versus 10 mg RSV, 10 ml of BSA solution, 4 h of stirring time; (*c*) 50 mg PLGA versus 10 ml of BSA solution, 4 h of stirring time; (*d*) 50 mg PLGA versus 9 mg RSV, 10 ml of BSA solution.

The effects of formulation variables on particle size or EE% are shown in figure 1. The most optimum volume ratio of organic and aqueous phase is 1 : 2 with the smallest particle size of NPs (figure 1*b*). And highly viscous organic phases produce larger particles due to the higher shear forces required to break the droplets [30,31]. The amount of RSV (figure 1*c*) and stirring time (figure 1*d*) had significant effects on EE% and DL%. The excessive RSV might contribute to the lessening EE% by impeding the whole stability of the formed RSV-PLGA-NPs when PLGA reached saturation [32,33]. This indicated that there was an optimal amount of RSV to reach the maximum EE% and DL% for RSV-PLGA-NPs. Stirring time should not be too long, as the drug would release from the nanoparticles. Besides, the stirring time could not be too short, which may decrease the stability of the nanoparticles and increase the toxicity *in vivo* due to residual organic solvent.

**Figure 2.**
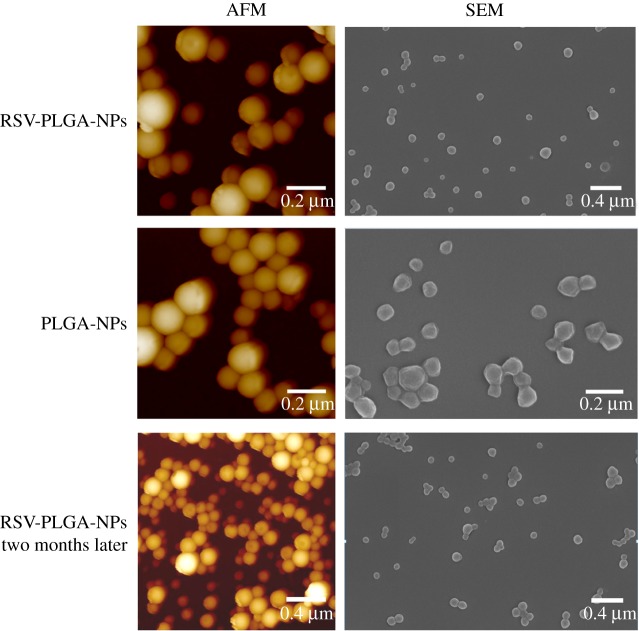
The images of SEM and AMF for RSV-PLGA-NPs and PLGA-NPs.

Considering the advantages of small particle size and high EE in drug delivery, the final formulation was typically determined as 50 mg of PLGA, 9 mg of RSV, 10 ml BSA solution and 4 h of stirring time. The resulting size of RSV-PLGA-NPs was 176.1 nm with PDI of 0.152 and that of empty NPs was 190 nm with PDI of 0.09 (figure 1*b* and table 1). The results showed that the molecule of RSV did not increase the size of RSV-PLGA-NPs. It may be because RSV was physically embedded in the nanoparticle cavities and distributed on its surface or core [34]. Initially, the nanoparticles showed a spherical shape with a low tendency of agglomeration and a slightly porous surface (figure 2) [35]. Their sizes observed by SEM and AFM were around 100 nm smaller than DLS-calculated size, which may be associated with the flattening of particles upon sampling [36]. The former only denoted the size of the core of the nanocarriers, while the latter one meant the hydrodynamic diameter [21,27]. The EE% and DL% of RSV-PLGA-NPs were determined to be 97.25% and 14.9%. The optimized method could ensure that EE% reached 90% or more each time the nanoparticles were made, showing an excellent drug entrapment capacity.

**Table 1.** The average size, PDI and zeta potential of nanoparticles (*n* = 3).

groups	PLGA-NPs	RSV-PLGA-NPs	RSV-PLGA-NPs two months later
size average (nm)	190	176.1	181.6
PDI	0.09	0.152	0.125
zeta potential (mV)	−20.3	−22.6	−23.1

Of note, zeta potential of RSV-PLGA-NPs and PLGA-NPs were negatively charged with values of −22.6 and −20.3, respectively. The zeta potential is an important indicator of the stability of colloidal dispersions, which can resist aggregation from each other [37]. The higher the zeta potential (positive or negative) is, the more stable the system is [38]. The absolute zeta potential greater than 20 mV suggested a high colloidal stability of RSV-PLGA-NPs due to the electrostatic repulsion [39]. In order to further test the stability of the RSV-PLGA-NPs, its particle size (figure 1*b*), zeta potential, PDI (table 1) and EE% (data not shown) after storage for two months were measured. The significant changes in particle size, PDI, zeta potential and EE% of nanoparticles did not occur. The physical stability of RSV-PLGA-NPs was shown to be fine for a short-term storage survey. Meanwhile, this can also maintain the stability of RSV to a certain extent.

### 3.2. Stability study

RSV-PLGA-NPs showed miniscule variation in the formulation parameters during six months of storage at the stability conditions of 4°C and protected from light (table 2). It meant that the NPs were stable enough with high encapsulation ability. After exposure to ultraviolet light for an hour, the bioactivity of RSV in RSV-PLGA-NPs was shown in cell experiments (figures 7 and 8). RSV-PLGA-NPs are more efficient in alleviating lipogenesis, promoting lipolysis and reducing hepatocellular proliferation than free RSV due to their enhanced stability, water solubility and bioactivity. It indicated that NPs could protect the RSV's bioactivity from isomerization due to UV light.

**Table 2.** The stability study of RSV-PLGA-NPs (*n* = 3).

time (month)	average size (nm)	PDI	zeta potential (mV)	EE%
0	178.2	0.114	−25.6	96.45%
1	179.3	0.126	−26.1	96.43%
3	179.8	0.107	−24.5	94.39%
6	178.5	0.112	−25.7	94.28%

### 3.3. FTIR spectroscopy

**Figure 3.**
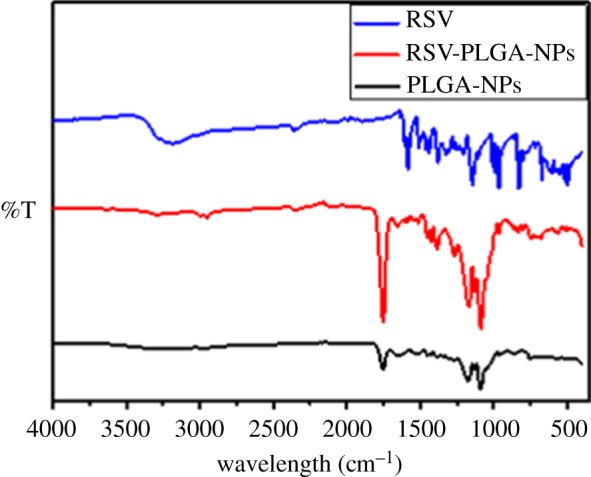
FTIR spectra of RSV, RSV-PLGA-NPs and PLGA-NPs (drug-free NPs).

RSV, RSV-PLGA-NPs and PLGA-NPs were characterized by FTIR spectra which were shown in figure 3. The RSV powder, lyophilized RSV-PLGA-NPs and lyophilized PLGA-NPs were measured with an ATR attachment of FTIR spectroscopy. ATR-FTIR spectroscopy is a versatile tool for the study of nanomaterial surfaces that can be used in either a qualitative or quantitative way [40]. The characteristic absorption peak of PLGA-NPs at 1750 cm^−1^ implied unconjugated carbonyl (C=O) stretching. RSV displayed its characteristic absorption bands at 3290 cm^−1^ for O–H stretching because of the alcoholic group, 964.7 cm^−1^ for *trans*-olefinic bond, 1583 cm^−1^ for C=C stretching of the aromatic ring, 1144 cm^−1^ for C–O stretching, respectively.

The RSV-PLGA-NPs performed all characteristic absorption peaks of PLGA-NPs; however, the characteristic peak of RSV that RSV-PLGA-NPs performed was not obvious, which was mainly because the ATR-FTIR spectroscopy only detected the information of nanomaterial surfaces while the RSV was mostly encapsulated in the core of RSV-PLGA-NPs. Even though there was some RSV on the surface of NPs, the content of RSV was too negligible to be detected when compared with the content of PLGA. The results also proved that there was no potential chemical reaction between RSV and any other formulation ingredients [41]. It confirmed that RSV was mostly contained within the nucleus of the nanoparticles, which could further avoid the isomerization caused by light to facilitate RSV's stability and biological activities.

### 3.4. Release kinetics *in vitro*

**Figure 4.**
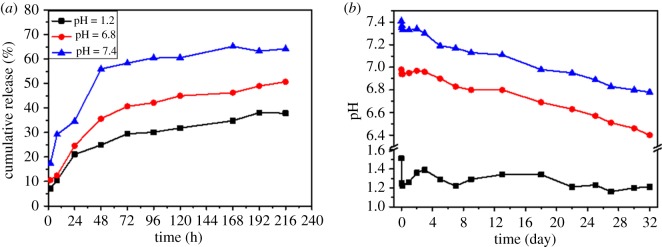
(*a*) *In vitro* release of RSV from RSV-PLGA-NPs in pH 1.2, pH 6.8 and pH 7.4, respectively, over a period of 216 h (9 days). (*b*) *In vitro* degradation of RSV-PLGA-NPs in pH 1.2, in pH 6.8 and in pH 7.4, respectively, over a period of 32 days.

The release profiles of RSV from RSV-PLGA-NPs were determined by imitating digestion conditions in gastric, intestinal and intravenous juice, respectively. As shown in figure 4*a*, RSV from RSV-PLGA-NPs showed the initial fast release with following delayed release without any burst release. After 8 h, at pH 7.4, the release of RSV reached 29.21%, at pH 6.8 reached 12.49% and at pH 1.2 it dropped to 10.56%. At the time point of 216 h, RSV-PLGA-NPs still have more than 30% of drug that has not been released. The results may suggest that RSV-PLGA-NPs could achieve a sustained and slow release process, which allowed the majority of RSV to be retained in NPs when transporting through the gastrointestinal tract [36], according to the lower release under acidic pH conditions compared with pH 7.4.

The absence of burst release may be attributed to lack of pristine RSV molecules at NPs' surface [25] and indicated that diffusion kinetics, which governs the burst phase [42], were independent of the media conditions [43]. The different release profiles of pH 7.4 were obtained, owing to the rate of release at pH 7.4 was faster than the other two. It has been known that release from high MW PLGA (e.g. 31 kDa) microsphere typically occurs via a combination of diffusion and erosion [42]. It indicated that the acidic pH affected the rate of release by affecting the rate of NPs’ degradation, which could accelerate the release of drugs [44,45].

### 3.5. Degradation kinetics *in vitro*

In order to validate whether the degradation of RSV-PLGA-NPs affected the release of RSV, *in vitro* degradation of RSV-PLGA-NPs was determined (figure 4*b*). As we can see, the most significant change of pH occurred under the condition of pH 7.4, in which pH dropped from 7.41 to 6.78, followed by conditions of pH 6.8 and 1.2. Fortunately, the results of the degradation of RSV-PLGA-NPs were consistent with the previous speculation that the pH conditions would change the rate of NPs' degradation. Besides, NPs’ degradation would affect the rate of release.

It is widely established that PLGA degradation in aqueous environment starts with water uptake and the random hydrolysis of the ester bonds in the polymer backbone [46]. Each hydrolyzed ester linkage forms one hydroxyl and one carboxylic acid group, which leads to the production of acidic oligomers [47]. These acidic oligomers are finally hydrolyzed to lactic and glycolic acids [47]. Therefore, the degradation of PLGA can be characterized based on changes in pH with the accumulation of acidic substances in the medium. The retention of the oligomeric degradation by-products within the microspheres, which results from their relative hydrophobicity, has been reported to impact the degradation mechanism [48]. The oligomeric units within NPs play an important role in NPs' degradation resulting in the formation of channels through which drug release occurs [49]. So it can be speculated that the pH conditions can affect the degree of uniformity of acidic oligomers in the nanoparticles [43]. RSV-PLGA-NPs incubated at higher pH were thought to be heterogeneous in pH as a result of pockets of acidic PLGA oligomers which cause non-uniform degradation creating a porous microstructure, which accelerates the release of RSV. The relative homogeneity in pH may lead to a relatively smooth appearance of NPs incubated at acidic pH, in which drug release slowed down. Of course, these guesses require a lot of research to confirm; we will do it later.

### 3.6. Cellular uptake

**Figure 5.**
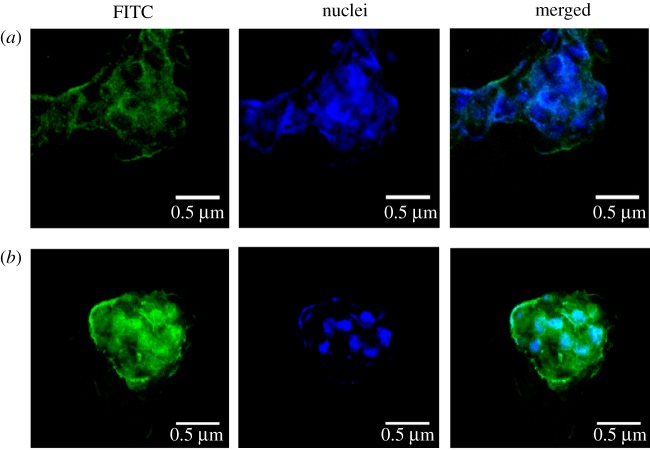
The fluorescent staining of HepG2 cells treated with FITC-PLGA-NPs for 2 h (*a*) and 8 h (*b*).

The fluorescent staining of HepG2 cells treated with FITC-labelled RSV-PLGA-NPs is shown in figure 5. RSV-PLGA-NPs could be internalized into the cells. NPs (green) co-localized with the cell nucleus (blue), taking on a light green upon overlay. The intracellular fluorescence intensity was weak when incubated for 2 h. But the fluorescence intensity became apparently strong after 8 h. This demonstrated that RSV-PLGA-NPs had a good cellular internalization and cytosolic delivery ability.

### 3.7. *In vitro* cytotoxicity assay

**Figure 6.**
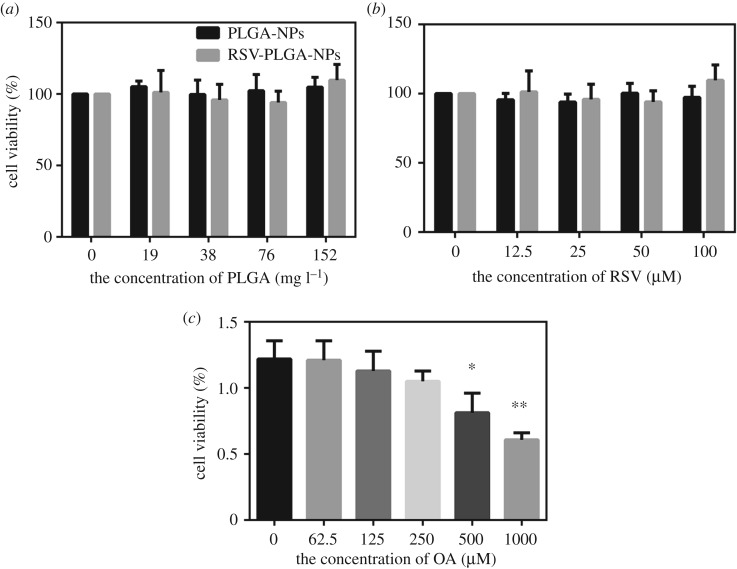
Cell viability of HepG2 cells treated with free RSV (*a*), PLGA-NPs (*b*), RSV-PLGA-NPs (*a,b*) and OA (*c*), respectively. In addition, the group 0 is the control (mean ± s.d., *n* = 3). *p < 0.05, **p < 0.01.

To avoid any misinterpretation because of cytotoxicity, the toxicity of RSV, PLGA-NPs, RSV-PLGA-NPs and OA to HepG2 cells should be tested. Their impacts on cell viability were shown in figure 6. There is no significant difference when RSV-PLGA-NPs and the free drug (RSV) were at the RSV concentration of 12.5 to 100 µM compared with the control group (the group 0), showing that the drug concentration in this range was non-toxic to cells for subsequent experiments (figure 6*b*). PLGA-NPs also had no toxic to cells in the suitable drug concentrations (figure 6*a*). The concentrations of RSV-PLGA-NPs were the same between these two figures. Consistent with the previous report [21], OA caused a dose-dependent cytotoxicity in HepG2 cells (figure 6*c*). Both 500 and 1000 µM of OA were toxic to cells, which affected the choice of OA's concentration we used in following experiments.

### 3.8. Oleic acid-induced hepatic steatosis

**Figure 7.**
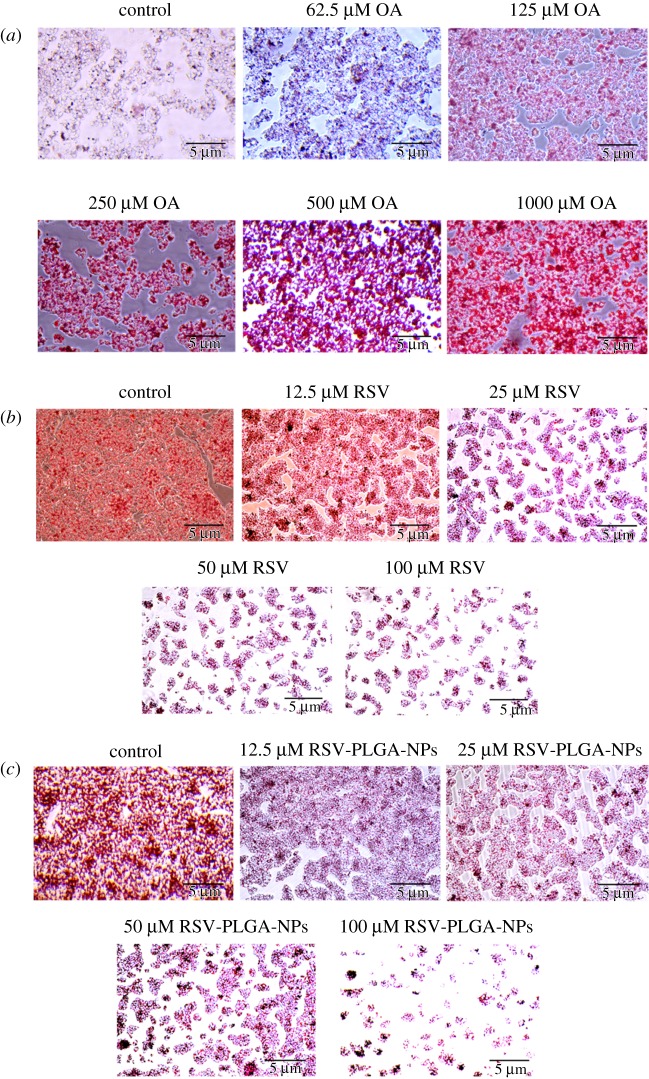
Oil Red O staining. (*a*) The effect of OA on hepatocyte steatosis in HepG2 cells. The effects of RSV (*b*) and RSV-PLGA-NPs (*c*) on TG accumulation in HepG2 induced by OA.

HepG2 cells were incubated with a concentration of OA for 24 h to build a model of NAFLD *in vitro* [21], and the result of the intracellular accumulation of TG was tested by Oil Red O staining. OA, a saturated fatty acid, is the main free fatty acids in the human body [50] and has been widely employed to induce the steatosis *in vitro* [51]. OA has less apoptotic nature and more steatogenic to hepatic cells than other fatty acids such as palmitic acid (PA) [52]. It could be seen that OA caused a concentration-dependent increase in accumulation of lipid (figure 7*a*). According to the significant induction in lipid accumulation [21], 250 µM of OA had been chosen as the optimal concentration, given the high dose of OA may increase the cytotoxicity (figure 6*c*). This concentration would be applied to subsequent experiments.

The steatotic HepG2 cells were induced with the same concentration of OA (250 µM) and then incubated with different concentrations of RSV and RSV-PLGA-NPs for 24 h, respectively. According to the results of figure 7*b,c*, RSV and RSV-PLGA-NPs could decrease the intracellular TG when compared to the control group. Less lipid accumulated as the drug concentration increased, which indicated that the drug's ability to inhibit lipid accumulation was dose-dependent. Furthermore, RSV-PLGA-NPs exposed to ultraviolet light for an hour still have the anti-adipogenic effect, which illustrated that RSV-PLGA-NPs had a great protective effect on the bioactivity of RSV.

The NAFLD has four stages and the simple steatosis has been regarded as the first stage in the development of NAFLD [53,54]. Lipid accumulation is the hallmark of NAFLD, a disease in which excessive lipid accumulates in hepatocytes, which has no relation to the alcohol consumption [4]. Therefore, it can be initially proved that RSV can at least prevent and treat the NAFLD at the first stage.

### 3.9. Quantitative assessment of triglycerides

**Figure 8.**
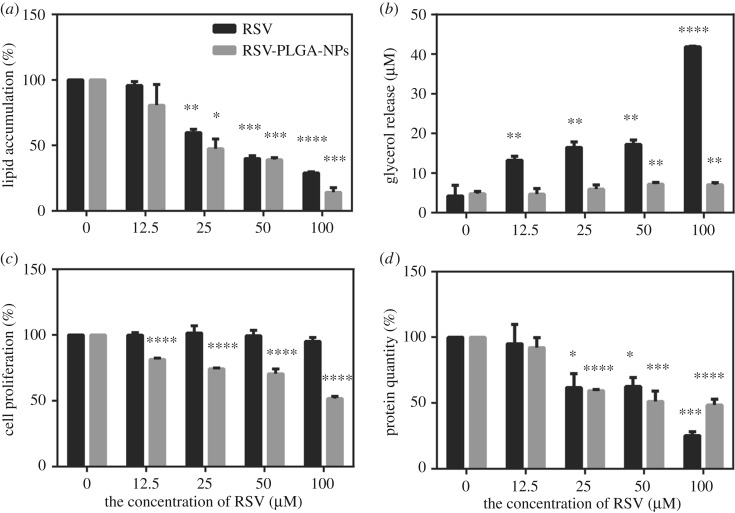
(*a*) The effect of RSV and RSV-PLGA-NPs on adipogenesis in OA-induced HepG2 cells. (*b*) The effect of RSV and RSV-PLGA-NPs on adipolysis in OA-induced HepG2 cells, characterized by glycerol release. The effects of RSV and RSV-PLGA-NPs on cell proliferation before (*c*) and after (*d*) induction with OA in HepG2. In addition, the group 0 is the control group (mean ± s.d., *n* = 3). *p < 0.05, **p < 0.01, ***p < 0.001, ****p < 0.0001.

As shown in figure 8*a*, RSV and RSV-PLGA-NPs inhibited lipid accumulation in a dose-dependent manner. When the concentration of RSV was more than 25 µM, both naked RSV and RSV-PLGA-NPs significantly inhibit lipid accumulation compared with the control group. RSV-PLGA-NPs remarkably attenuated lipid accumulation by 80.77% (100 µM) and free RSV reduce it by 71.07% (100 µM). Thus, RSV-PLGA-NPs more effectively inhibit lipid accumulation than RSV. In other words, these results also indicated that RSV-PLGA-NPs could enhance the bioactivity of RSV.

### 3.10. Quantitative assessment of glycerol

To assess whether RSV and RSV-PLGA-NPs increased lipolysis, the steatotic HepG2 cells were incubated with RSV and RSV-PLGA-NPs, respectively, for 24 h followed by the determination of glycerol release in the culture medium. As shown in figure 8*b*, free RSV and RSV-PLGA-NPs significantly increased glycerol release when compared to the control. This result was consistent with the result of inhibiting the accumulation of lipid. But the glycerol content was tested higher than control when the concentration of RSV-PLGA-NPs was high, which might be because the PLGA carrier particle itself could absorb glycerol. PLGA is soluble in some non-polar organic solvents [26]. According to the principle of similar compatibility, glycerol, a non-polar organic substance, is more easily absorbed by PLGA than soluble in water. Among these, RSV and RSV-PLGA-NPs showed an ability to promote lipolysis.

### 3.11. Cell proliferation

To explore whether RSV and RSV-PLGA-NPs had an inhibitory effect on cell proliferation, cell proliferation before and after induction were determined. As was shown in figure 7*c*, free drug (RSV) had no effect on cell proliferation, while drug-loaded nanoparticles (RSV-PLGA-NPs) had a significant inhibitory effect on cell proliferation (compared with the control group). This may be due to the absorbing of serum (serum can promote cell proliferation) from the medium by nanoparticles. As the concentration of nanoparticles increased, more serum was absorbed by the PLGA, and a greater degree of inhibition was made by the nanoparticles. As was demonstrated in figure 7*d*, RSV and RSV-PLGA-NPs inhibited hepatocellular proliferation after the induction in a dose-dependent manner. They had a similar inhibitory effect on cells after induction.

In summary, RSV-PLGA-NPs played a role in reducing hepatocellular proliferation. However, free RSV had the function only after induction. One of the reasons might be the encapsulation of the nano-shell enhanced the bioactivity of the drug. It is known that the increased hepatocellular proliferation and turnover in the setting of steatosis may play important roles in the progression and complications of NAFLD [55]. So the RSV’s function of reducing hepatocellular proliferation may provide a new way to treat NAFLD.

## 4. Conclusion

In summary, the optimized O/W emulsion technique in this study allowed the reproducible and momentary formation of nanometric (176.1 nm), stable and spherical nanoparticles, which exhibited a remarkable EE% (97.25%) and DL% (14.9%) for RSV with an adjustable dosage ratio. And RSV-PLGA-NPs could achieve a sustained and slow release process especially in acidic pH conditions, which allowed the majority of RSV to be retained in NPs when simulating transporting through the gastrointestinal tract. In addition, RSV-PLGA-NPs were more efficient in alleviating lipogenesis, promoting lipolysis and reducing hepatocellular proliferation than free RSV due to its improved stability, water solubility and bioactivity. In other words, RSV-PLGA-NPs can enhance the stability, solubility and bioactivity significantly to treat NAFLD. In particular, the use of nanotechnology to encapsulate RSV and the inhibitory effects of RSV on cell proliferation have rarely been reported for the treatment of NAFLD. Therefore, this research may provide a new way to solve the existing problems such as the poor aqueous solubility, chemical instability and intestinal metabolism of RSV, and no accepted pharmacologic therapies for NAFLD currently exist. Overall, RSV-PLGA-NPs have a promising potential for NAFLD therapy, and there is still a lot of research needing to be done *in vivo*, if it is to be applied to treatment.

## Supplementary Material

Figure S1

## Supplementary Material

Figure S2

## Supplementary Material

Figure S3

## Supplementary Material

Figure S4

## Supplementary Material

Figure S5

## Supplementary Material

Figure S6

## Supplementary Material

Figure S7

## Supplementary Material

Figure S8

## Supplementary Material

Table S1

## Supplementary Material

Table S2

## Data accessibility

The datasets supporting this article have been uploaded as part of the electronic supplementary material and also deposited at the online figshare repository. (https://figshare.com/s/588ff5ef7ee0e2ccf903)

## References

[1] Berman AY, Motechin RA, Wiesenfeld MY, Holz MK 2017 The therapeutic potential of resveratrol: a review of clinical trials. NPJ Precis. Oncol. 1, 35 (10.1038/s41698-017-0038-6) 28989978 PMC5630227

[2] Chachay VS et al 2014 Resveratrol does not benefit patients with nonalcoholic fatty liver disease. Clin. Gastroenterol. Hepatol. 12, 2092-2103. (10.1016/j.cgh.2014.02.024) 24582567

[3] Woods CP, Hazlehurst JM, Tomlinson JW 2015 Glucocorticoids and non-alcoholic fatty liver disease. J. Steroid Biochem. Mol. Biol. 154, 94–103. (10.1016/j.jsbmb.2015.07.020) 26241028

[4] Cusi K 2012 Role of obesity and lipotoxicity in the development of nonalcoholic steatohepatitis: pathophysiology and clinical implications. Gastroenterology 142, 711–725. (10.1053/j.gastro.2012.02.003) 22326434

[5] Said A 2013 Non-alcoholic fatty liver disease and liver transplantation: outcomes and advances. World J. Gastroenterol. 19, 9146–9155. (10.3748/wjg.v19.i48.9146) 24409043 PMC3882389

[6] Schuppan D, Gorrell MD, Klein T, Mark M, Afdhal NH 2010 The challenge of developing novel pharmacological therapies for non-alcoholic steatohepatitis. Liver Int. 30, 795–808. (10.1111/j.1478-3231.2010.02264.x) 20624207

[7] Beaton MD 2012 Current treatment options for nonalcoholic fatty liver disease and nonalcoholic steatohepatitis. Can. J. Gastroenterol. 26, 353–357. 22720278 10.1155/2012/725468 PMC3378283

[8] Vajro P, Lenta S, Pignata C, Salerno M, D'Aniello R, De Micco I, Paolella G, Parenti G 2012 Therapeutic options in pediatric non alcoholic fatty liver disease: current status and future directions. Ital. J. Pediatr. 38, 55 (10.1186/1824-7288-38-55) 23075296 PMC3534557

[9] Mouzaki M, Allard JP 2012 The role of nutrients in the development, progression, and treatment of nonalcoholic fatty liver disease. J. Clin. Gastroenterol. 46, 457–467. (10.1097/MCG.0b013e31824cf51e) 22469640

[10] Rauf A, Imran M, Suleria HAR, Ahmad B, Peters DG, Mubarak MS 2017 A comprehensive review of the health perspectives of resveratrol. Food Funct. 8, 4284–4305. (10.1039/c7fo01300k) 29044265

[11] Öztürk E, Arslan AKK, Yerer MB, Bishayee A 2017 Resveratrol and diabetes: a critical review of clinical studies. Biomed. Pharmacother. 95, 230–234. (10.1016/j.biopha.2017.08.070) 28843911

[12] Fernández-Quintela A, Milton-Laskibar I, González M, Portillo MP 2017 Antiobesity effects of resveratrol: which tissues are involved? Ann. N. Y. Acad. Sci. 1403, 118–131. (10.1111/nyas.13413) 28796895

[13] Singh G, Pai RS 2014 Optimized PLGA nanoparticle platform for orally dosed *trans*-resveratrol with enhanced bioavailability potential. Expert Opin. Drug Deliv. 11, 647–659. (10.1517/17425247.2014.890588) 24661109

[14] Charytoniuk T, Drygalski K, Konstantynowicz-Nowicka K, Berk K, Chabowski A 2017 Alternative treatment methods attenuate the development of NAFLD: a review of resveratrol molecular mechanisms and clinical trials. Nutrition 34, 108–117. (10.1016/j.nut.2016.09.001) 28063505

[15] Heeboll S et al. 2016 Placebo-controlled, randomised clinical trial: high-dose resveratrol treatment for non-alcoholic fatty liver disease. Scand. J. Gastroenterol. 51, 456–464. (10.3109/00365521.2015.1107620) 26784973

[16] Zhang Y et al. 2015 Resveratrol improves hepatic steatosis by inducing autophagy through the cAMP signaling pathway. Mol. Nutr. Food Res. 59, 1443–1457. (10.1002/mnfr.201500016) 25943029

[17] Choi YJ, Suh HR, Yoon Y, Lee KJ, Kim DG, Kim S, Lee BH 2014 Protective effect of resveratrol derivatives on high-fat diet induced fatty liver by activating AMP-activated protein kinase. Arch. Pharm. Res. 37, 1169–1176. (10.1007/s12272-014-0347-z) 24633463

[18] Heeboll S et al. 2015 Effect of resveratrol on experimental non-alcoholic steatohepatitis. Pharmacol. Res. 95–96, 34–41. (10.1016/j.phrs.2015.03.005) 25814186

[19] Francioso A, Mastromarino P, Masci A, d'Erme M, Mosca L. 2014 Chemistry, stability and bioavailability of resveratrol. Med. Chem. (Shariqah (United Arab Emirates)) 10, 237–245. 10.2174/15734064113096660053 24329932

[20] Zupancic S, Lavric Z, Kristl J 2015 Stability and solubility of *trans*-resveratrol are strongly influenced by pH and temperature. Eur. J. Pharm. Biopharm. 93, 196–204. (10.1016/j.ejpb.2015.04.002) 25864442

[21] Liu Y, Wu X, Mi Y, Zhang B, Gu S, Liu G, Li X 2017 PLGA nanoparticles for the oral delivery of nuciferine: preparation, physicochemical characterization and *in vitro*/*in vivo* studies. Drug Deliv. 24, 443–451. (10.1080/10717544.2016.1261381) 28165858 PMC8241190

[22] Wang H et al. 2017 New generation nanomedicines constructed from self-assembling small-molecule prodrugs alleviate cancer drug toxicity. Cancer Res. 77, 6963–6974. (10.1158/0008-5472.CAN-17-0984) 29055017

[23] Chen Q, Yang Y, Lin X, Ma W, Chen G, Li W, Wang X, Yu Z 2018 Platinum(iv) prodrugs with long lipid chains for drug delivery and overcoming cisplatin resistance. Chem. Commun. 54, 5369–5372. (10.1039/c8cc02791a) 29744485

[24] Lin YF, Lee YH, Hsu YH, Chen YJ, Lin YF, Cheng FY, Chiu HW 2017 Resveratrol-loaded nanoparticles conjugated with kidney injury molecule-1 as a drug delivery system for potential use in chronic kidney disease. Nanomedicine 12, 2741–2756. (10.2217/nnm-2017-0256) 28884615

[25] Vijayakumar MR et al 2013 Resveratrol loaded PLGA_D-α-tocopheryl polyethylene glycol 1000 succinate blend nanoparticles for brain cancer therapy. RSC Adv. 6, 74 254–74 268. (10.1039/C6RA15408E)

[26] Kapoor DN, Bhatia A, Kaur R, Sharma R, Kaur G, Dhawan S 2015 PLGA: a unique polymer for drug delivery. Ther. Deliv. 6, 41–58. (10.4155/tde.14.91) 25565440

[27] Gidwani B, Vyas A 2016 Formulation, characterization and evaluation of cyclodextrin-complexed bendamustine-encapsulated PLGA nanospheres for sustained delivery in cancer treatment. Pharm. Dev. Technol. 21, 161–171. (10.3109/10837450.2014.979945) 25391288

[28] Sun SB, Liu P, Shao FM, Miao QL 2015 Formulation and evaluation of PLGA nanoparticles loaded capecitabine for prostate cancer. Int. J. Clin. Exp. Med. 8, 19 670–19 681. PMC4694531 26770631

[29] Zhang X, Wang H, Zhang T, Zhou X, Wu B 2014 Exploring the potential of self-assembled mixed micelles in enhancing the stability and oral bioavailability of an acid-labile drug. Eur. J. Pharm. Sci. 62, 301–308. (10.1016/j.ejps.2014.06.008) 24956461

[30] Gaignaux A, Reeff J, Siepmann F, Siepmann J, De Vriese C, Goole J, Amighi K 2012 Development and evaluation of sustained-release clonidine-loaded PLGA microparticles. Int. J. Pharm. 437, 20–28. (10.1016/j.ijpharm.2012.08.006) 22903047

[31] Freitas S, Merkle HP, Gander B 2005 Microencapsulation by solvent extraction/evaporation: reviewing the state of the art of microsphere preparation process technology. J. Control Release 102, 313–332. (10.1016/j.jconrel.2004.10.015) 15653154

[32] Wei Z, Hao J, Yuan S, Li Y, Juan W, Sha X, Fang X 2009 Paclitaxel-loaded Pluronic P123/F127 mixed polymeric micelles: formulation, optimization and *in vitro* characterization. Int. J. Pharm. 376, 176–185. (10.1016/j.ijpharm.2009.04.030) 19409463

[33] Xin H et al. 2010 Enhanced anti-glioblastoma efficacy by PTX-loaded PEGylated poly(varepsilon-caprolactone) nanoparticles: *in vitro* and *in vivo* evaluation. Int. J. Pharm. 402, 238–247. (10.1016/j.ijpharm.2010.10.005) 20934500

[34] Danhier F, Ansorena E, Silva JM, Coco R, Le Breton A, Preat V 2012 PLGA-based nanoparticles: an overview of biomedical applications. J. Control Release 161, 505–522. (10.1016/j.jconrel.2012.01.043) 22353619

[35] Zhu W, Masaki T, Bae YH, Rathi R, Cheung AK, Kern SE 2006 Development of a sustained-release system for perivascular delivery of dipyridamole. J. Biomed. Mater. Res. B Appl. Biomater. 77, 135–143. (10.1002/jbm.b.30412) 16206204

[36] Siu FY, Ye S, Lin H, Li S 2018 Galactosylated PLGA nanoparticles for the oral delivery of resveratrol: enhanced bioavailability and *in vitro* anti-inflammatory activity. Int. J. Nanomed. 13, 4133–4144. (10.2147/IJN.S164235) PMC6049601 30038494

[37] Gossmann R, Langer K, Mulac D 2015 New perspective in the formulation and characterization of didodecyldimethylammonium bromide (DMAB) stabilized poly(lactic-co-glycolic acid) (PLGA) nanoparticles. PloS ONE 10, e0127532 (10.1371/journal.pone.0127532) 26147338 PMC4493066

[38] Carbone C, Martins-Gomes C, Caddeo C, Silva AM, Musumeci T, Pignatello R, Puglisi G, Souto EB 2018 Mediterranean essential oils as precious matrix components and active ingredients of lipid nanoparticles. Int. J. Pharm. 548, 217–226. (10.1016/j.ijpharm.2018.06.064) 29966744

[39] Chen Z, Tai Z, Gu F, Hu C, Zhu Q, Gao S 2016 Aptamer-mediated delivery of docetaxel to prostate cancer through polymeric nanoparticles for enhancement of antitumor efficacy. Eur. J. Pharm. Biopharm. 107, 130–141. (10.1016/j.ejpb.2016.07.007) 27393562

[40] Mudunkotuwa IA, Minshid AA, Grassian VH 2014 ATR-FTIR spectroscopy as a tool to probe surface adsorption on nanoparticles at the liquid-solid interface in environmentally and biologically relevant media. Analyst 139, 870–881. (10.1039/c3an01684f) 24350328

[41] Li P, Yang Z, Wang Y, Peng Z, Li S, Kong L, Wang Q 2015 Microencapsulation of coupled folate and chitosan nanoparticles for targeted delivery of combination drugs to colon. J. Microencapsul. 32, 40–45. (10.3109/02652048.2014.944947) 25198909

[42] Zolnik BS, Leary PE, Burgess DJ 2006 Elevated temperature accelerated release testing of PLGA microspheres. J. Control Release 112, 293–300. (10.1016/j.jconrel.2006.02.015) 16644055

[43] Zolnik BS, Burgess DJ 2007 Effect of acidic pH on PLGA microsphere degradation and release. J. Control Release 122, 338–344. (10.1016/j.jconrel.2007.05.034) 17644208

[44] Ponnusamy T, Lawson LB, Freytag LC, Blake DA, Ayyala RS, John VT 2012 *In vitro* degradation and release characteristics of spin coated thin films of PLGA with a ‘breath figure’ morphology. Biomatter 2, 77–86. (10.4161/biom.20390) 23507805 PMC3549860

[45] Holy CE, Dang SM, Davies JE, Shoichet MS 1999 *In vitro* degradation of a novel poly(lactide-co-glycolide) 75/25 foam. Biomaterials 20, 1177–1185. 10395386 10.1016/s0142-9612(98)00256-7

[46] Siepmann J, Gopferich A 2001 Mathematical modeling of bioerodible, polymeric drug delivery systems. Adv. Drug Deliv. Rev. 48, 229–247. 11369084 10.1016/s0169-409x(01)00116-8

[47] Makadia HK, Siegel SJ 2011 Poly lactic-co-glycolic acid (PLGA) as biodegradable controlled drug delivery carrier. Polymers 3, 1377–1397. (10.3390/polym3031377) 22577513 PMC3347861

[48] Fu K, Pack DW, Klibanov AM, Langer R 2000 Visual evidence of acidic environment within degrading poly(lactic-co-glycolic acid) (PLGA) microspheres. Pharm. Res. 17, 100–106. 10714616 10.1023/a:1007582911958

[49] Galeska I, Kim TK, Patil SD, Bhardwaj U, Chatttopadhyay D, Papadimitrakopoulos F, Burgess DJ 2005 Controlled release of dexamethasone from PLGA microspheres embedded within polyacid-containing PVA hydrogels. AAPS J. 7, E231–E240. (10.1208/aapsj070122) 16146344 PMC2751512

[50] Grishko V, Rachek L, Musiyenko S, Ledoux SP, Wilson GL 2005 Involvement of mtDNA damage in free fatty acid-induced apoptosis. Free Radic. Biol. Med. 38, 755–762. (10.1016/j.freeradbiomed.2004.11.023) 15721986

[51] Chiu HC, Kovacs A, Ford DA, Hsu FF, Garcia R, Herrero P, Saffitz JE, Schaffer JE 2001 A novel mouse model of lipotoxic cardiomyopathy. J. Clin. Invest. 107, 813–822. (10.1172/jci10947) 11285300 PMC199569

[52] Cui Y, Wang Q, Yi X, Zhang X 2016 Effects of fatty acids on CYP2A5 and Nrf2 expression in mouse primary hepatocytes. Biochem. Genet. 54, 29–40. (10.1007/s10528-015-9697-6) 26423681

[53] Michelotti GA, Machado MV, Diehl AM 2013 NAFLD, NASH and liver cancer. Nat. Rev. Gastroenterol. Hepatol. 10, 656–665. (10.1038/nrgastro.2013.183) 24080776

[54] Cohen J 2011 Understanding HIV latency to undo it. Science 332, 786 (10.1126/science.332.6031.786) 21566174

[55] Vansaun MN, Mendonsa AM, Lee Gorden D 2013 Hepatocellular proliferation correlates with inflammatory cell and cytokine changes in a murine model of nonalchoholic fatty liver disease. PloS ONE 8, e73054 (10.1371/journal.pone.0073054) 24039859 PMC3767686

