# Correction to ‘Resveratrol-loaded PLGA nanoparticles: enhanced stability, solubility and bioactivity of resveratrol for non-alcoholic fatty liver disease therapy’

**DOI:** 10.1098/rsos.182173

**Published:** 2019-01-16

**Authors:** S. Wan, L. Zhang, Y. Quan, K. Wei

*R. Soc. open sci.* **5**, 181457. (Published 1 November 2018). (doi:10.1098/rsos.181457)

This correction refers to an error in figure 6. The key to symbols for (*b*) was missing. The corrected figure and caption appear below.

**Figure 6.**
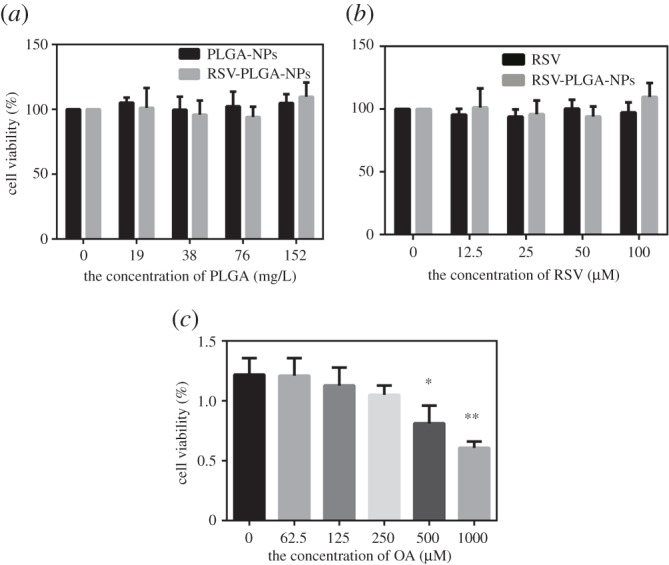
Cell viability of HepG2 cells treated with PLGA-NPs (*a*), free RSV (*b*), RSV-PLGA-NPs (*a,b*) and OA (*c*). In addition, the group 0 is the control (mean ± s.d., *n* = 3). *p < 0.05, **p < 0.01.

