# Supplementary figures and images for: Resveratrol-loaded PLGA nanoparticles: enhanced stability, solubility and bioactivity of resveratrol for non-alcoholic fatty liver disease therapy
Source: R Soc Open Sci. 2018 Nov 14;5(11):181457. doi: 10.1098/rsos.181457 (PMC6281916; doi:10.1098/rsos.181457)

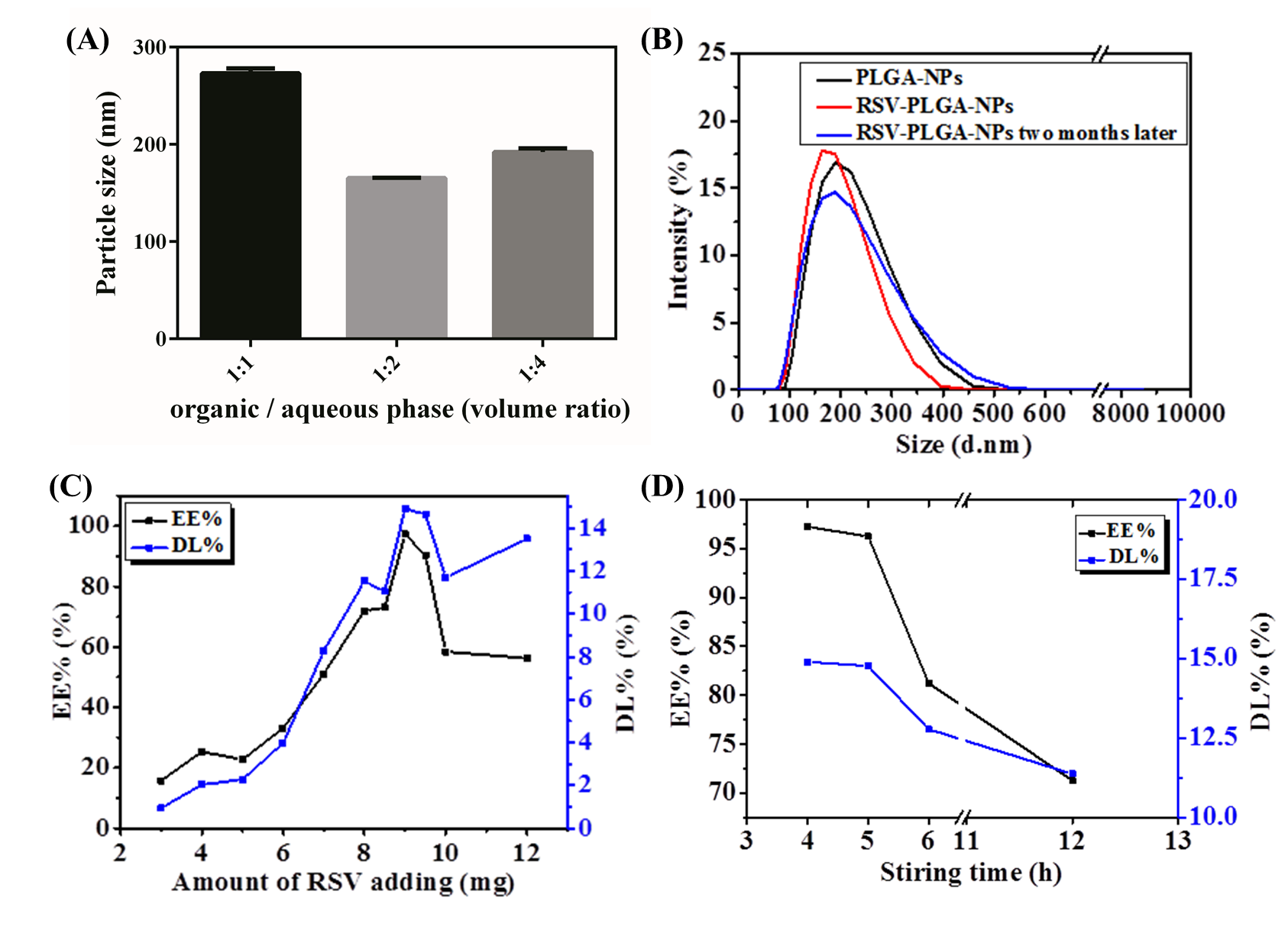

Supplement: Figure S1 [file rsos181457supp1.tif]

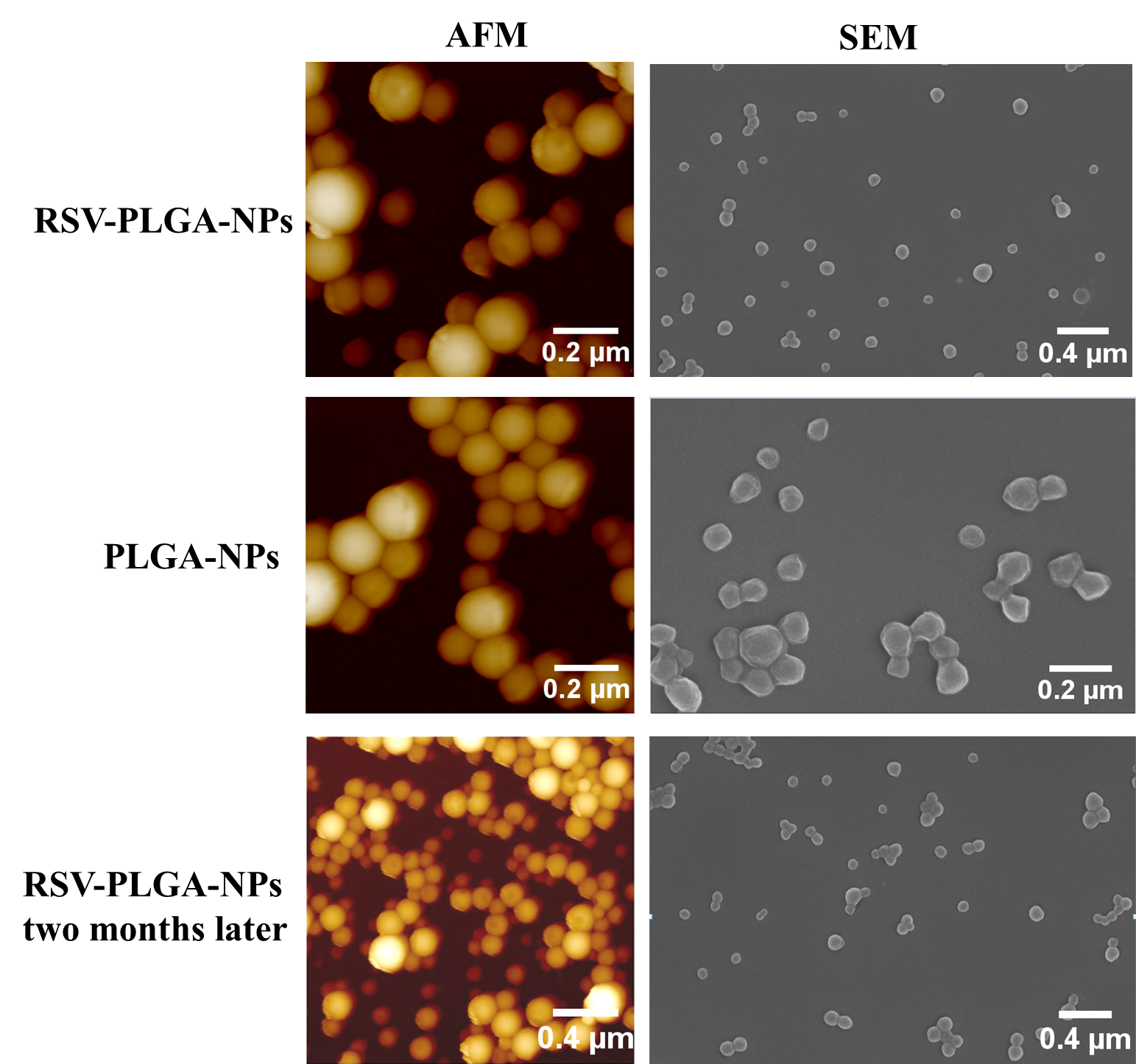

Supplement: Figure S2 [file rsos181457supp2.tif]

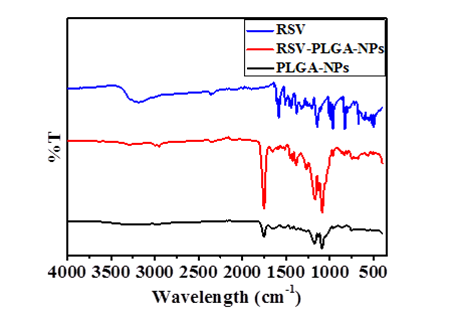

Supplement: Figure S3 [file rsos181457supp3.tif]

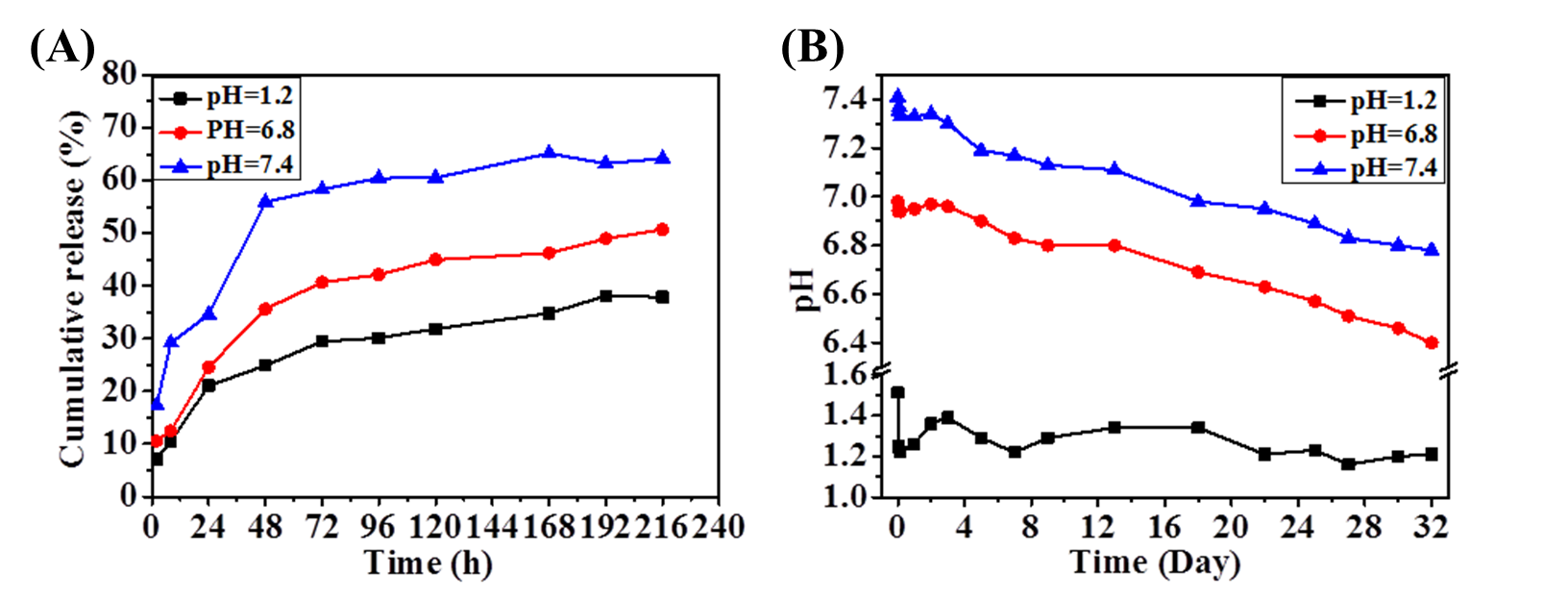

Supplement: Figure S4 [file rsos181457supp4.tif]

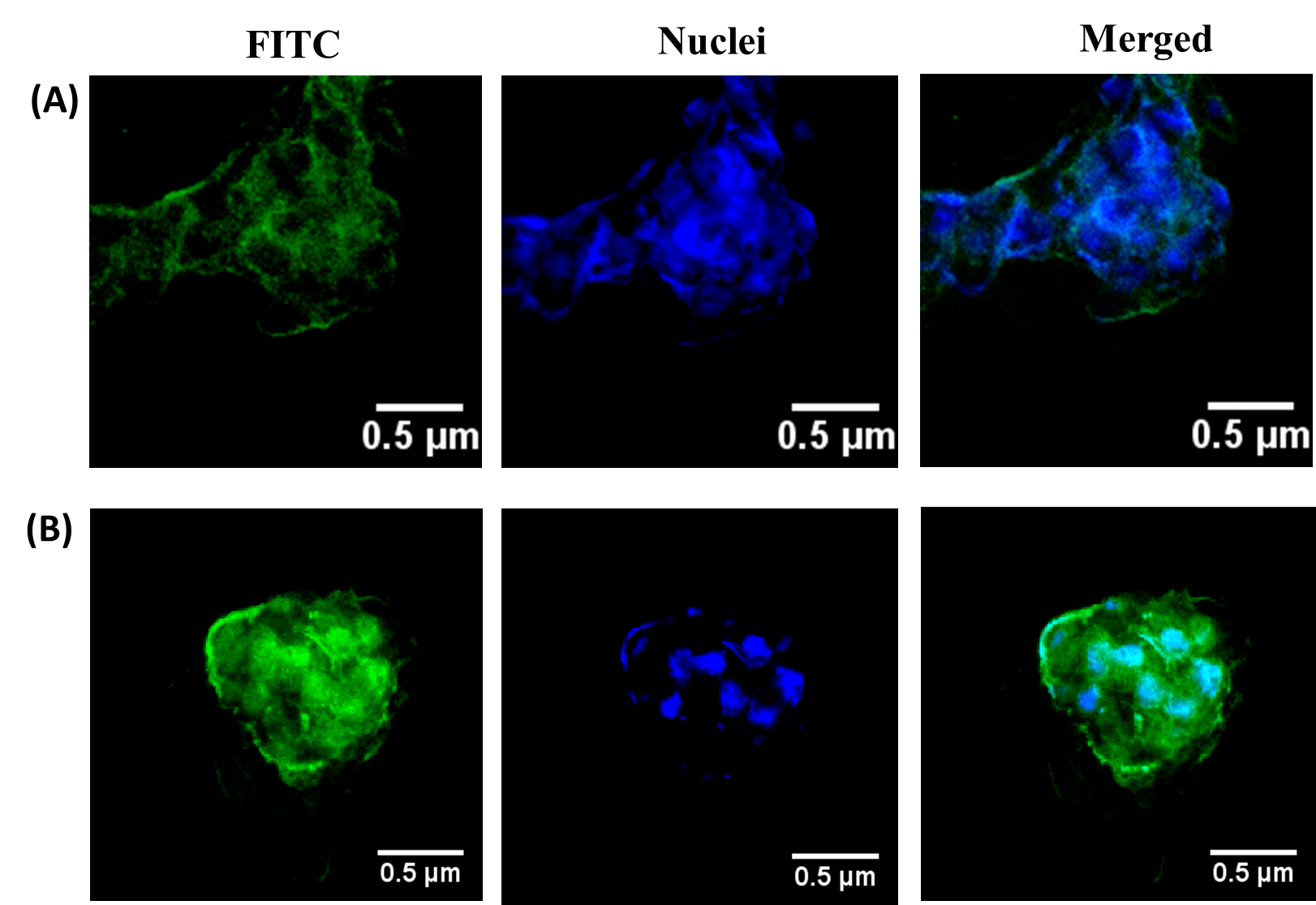

Supplement: Figure S5 [file rsos181457supp5.tif]

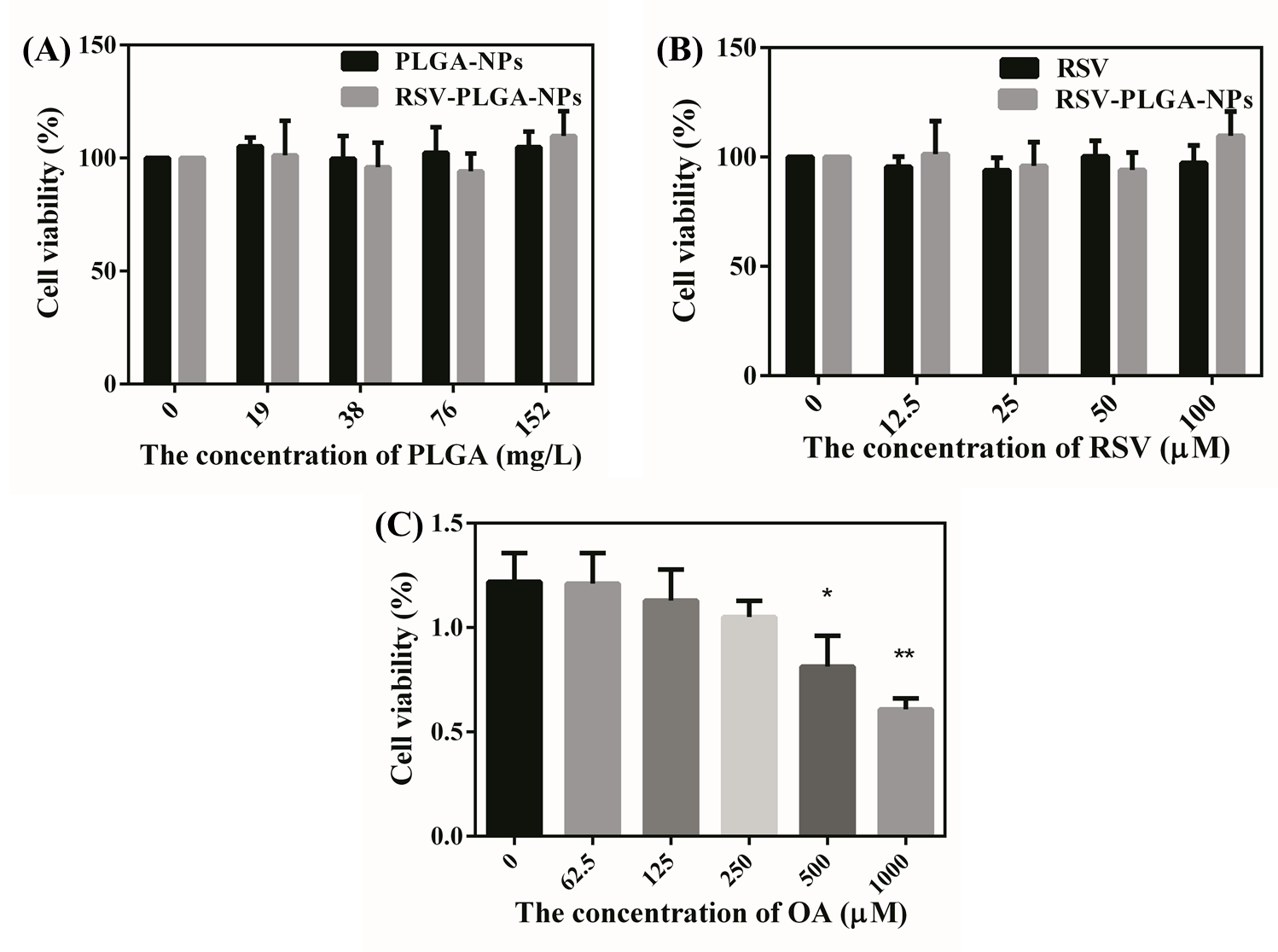

Supplement: Figure S6 [file rsos181457supp6.tif]

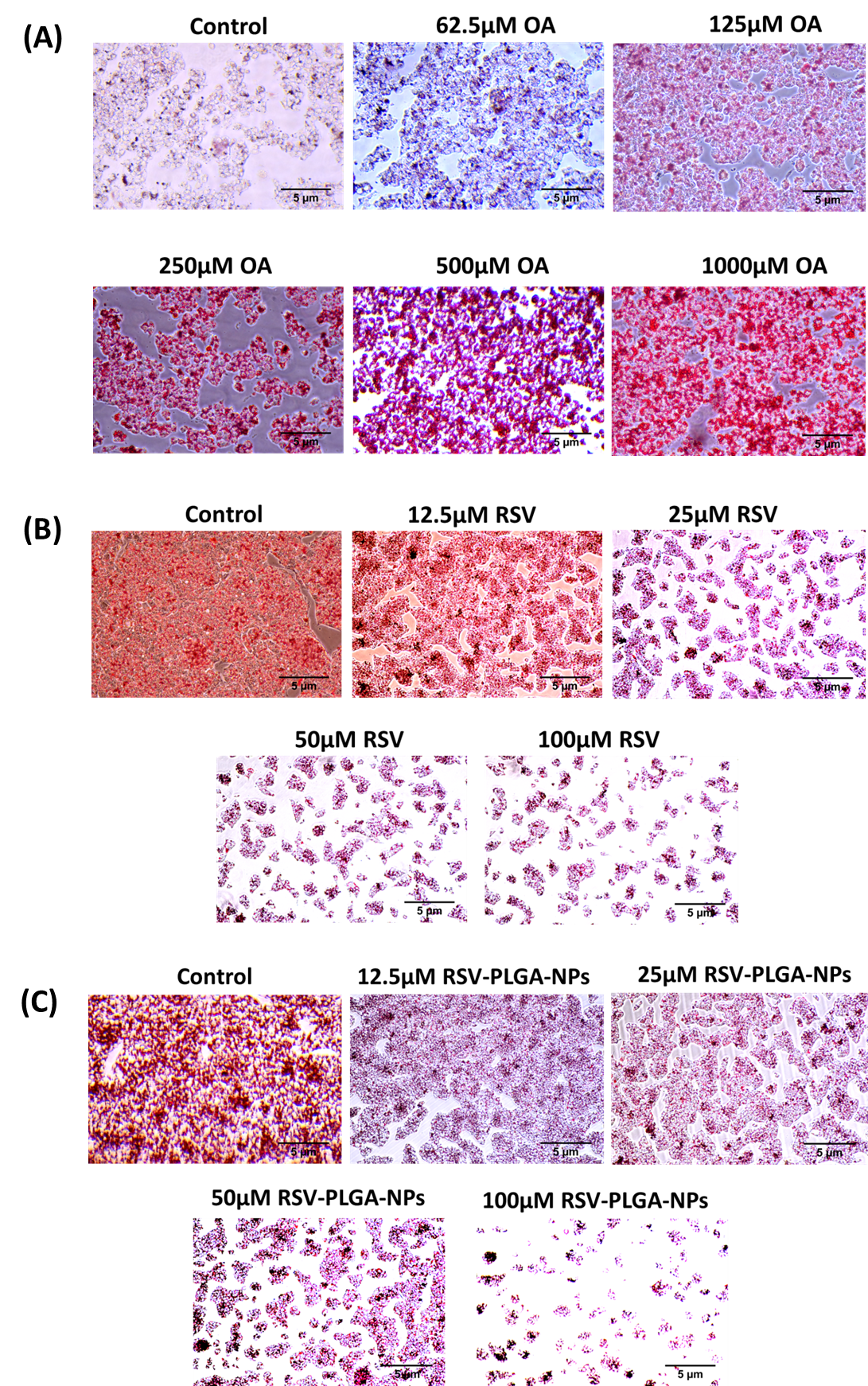

Supplement: Figure S7 [file rsos181457supp7.png]

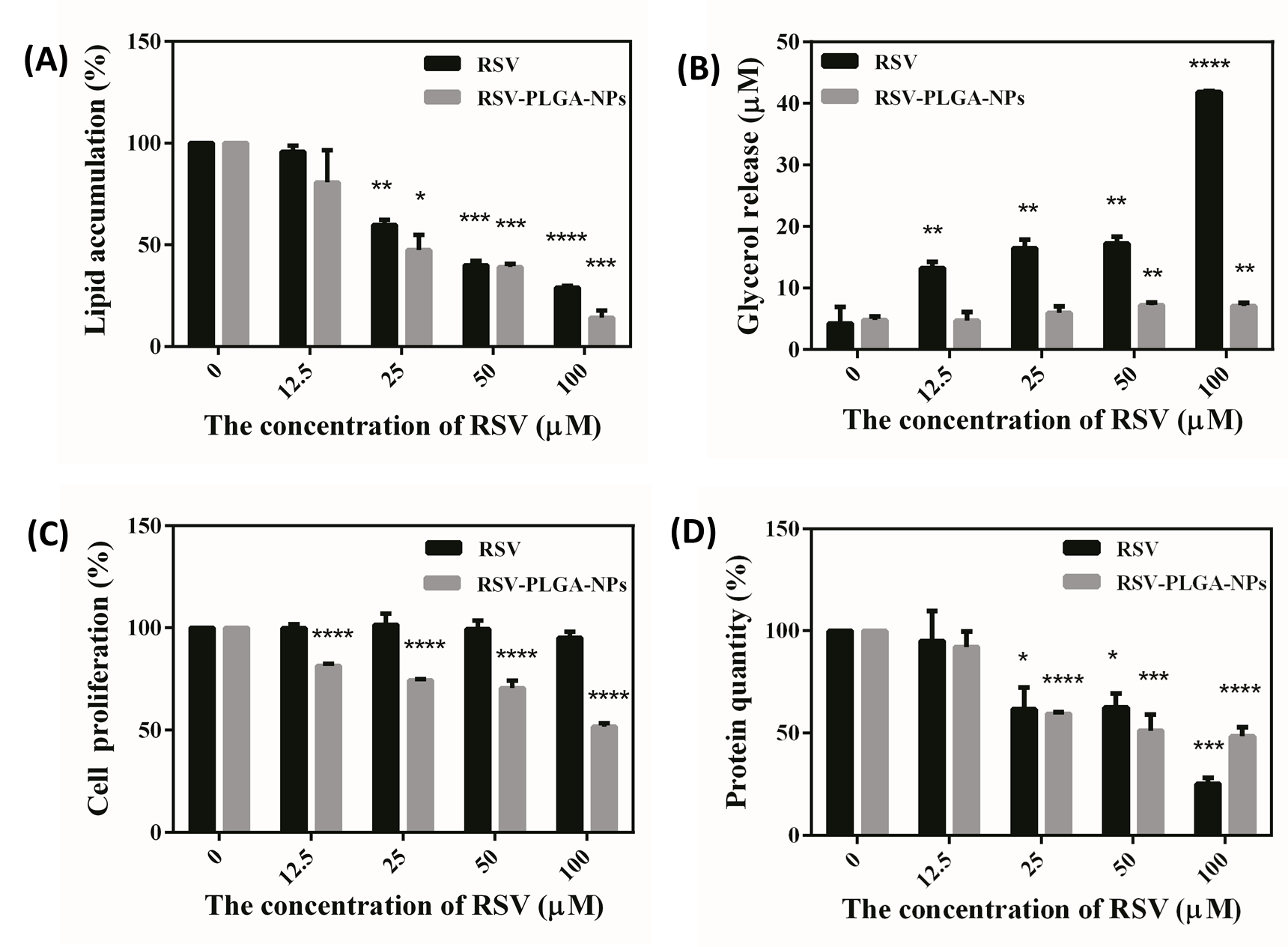

Supplement: Figure S8 [file rsos181457supp8.png]

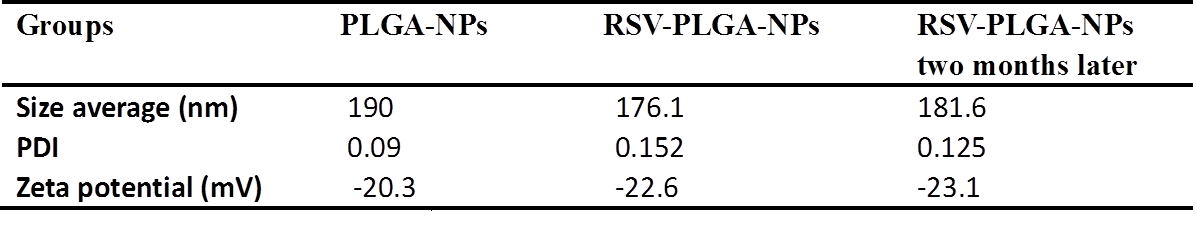

Supplement: Table S1 [file rsos181457supp9.tif]

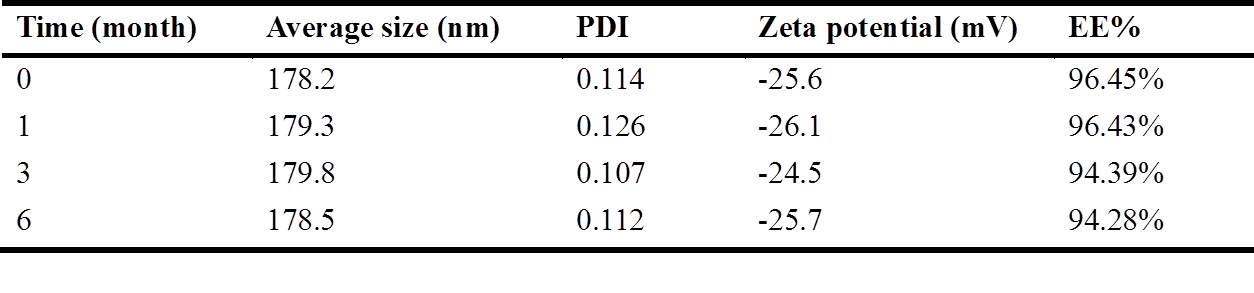

Supplement: Table S2 [file rsos181457supp10.tif]
